# An Intervention in Reading Disabilities Using a Digital Tool During the COVID-19 Pandemic

**DOI:** 10.3389/fpsyg.2022.862383

**Published:** 2022-05-06

**Authors:** Irene Cadime, Iolanda Ribeiro, Joana Cruz, Maria do Céu Cosme, Diana Meira, Fernanda Leopoldina Viana, Sandra Santos

**Affiliations:** ^1^Psychology Research Center, School of Psychology, University of Minho, Braga, Portugal; ^2^Psychology for Positive Development Research Center–CIPD, Lusíada University of Porto, Porto, Portugal; ^3^aPsi-Psychology Association of the University of Minho, Braga, Portugal; ^4^Research Centre on Child Studies, Institute of Education, University of Minho, Braga, Portugal; ^5^Centre for Research in Higher Education Policies, Matosinhos, Portugal

**Keywords:** reading disabilities, Tier 2 intervention, digital tool, remote intervention, COVID-19

## Abstract

In the last decade, ICT-based interventions for developing reading skills in children with reading disabilities have become increasingly popular. This study had three goals: (a) to assess the existence of gains in word reading, oral reading fluency and listening comprehension after a Tier 2 intervention using the digital tool “I’m still learning,” which was delivered partially in a remote modality during the COVID-19 pandemic; (b) to investigate whether the gains depended on the students’ gender, the number of sessions attended and the interventionist; and (c) to investigate parents’ perceptions about the suitability and perceived effects of the intervention. A single group design with pre-test and post-test was used. The intervention was delivered to second graders (*N* = 81) flagged as being at-risk for reading disabilities in a universal screening. The analyses showed significant gains in all three outcome variables after the intervention. The gains did not depend on students’ gender, number of intervention sessions attended or interventionist. Parents’ perceptions of the remote intervention were positive. The study findings highlight the potentialities of using technology-based interventions to foster reading skills and suggest that these may be especially useful during lockdowns.

## Introduction

Response to Intervention (RtI) models provide a line of action regarding assessment and intervention in several areas, including reading: evidence-based interventions are delivered, the effectiveness of those interventions is monitored, and the instruction is adjusted based on how a student responds (Fuchs and Fuchs, 2006; van Norman et al., 2020). Typically, RtI includes three Tiers: Tier 1, which includes universal evidence-based instruction; Tier 2, which encompasses more support for some students in addition to general instruction; and Tier 3 which involves a more intense and personalized intervention, often delivered individually, to only a few students (Gartland and Strosnider, 2020). Students enter Tier 2 when flagged in universal screenings that are used to identify students at-risk for reading disabilities (McAlenney and Coyne, 2011). In RtI models the Tier 2 intervention is delivered in small groups, usually ranging from 2 to 10 students (Balu et al., 2015), and is systematic and tailored to the students’ needs (Truckenmiller and Brehmer, 2021). Student progress is regularly monitored and those who fail to demonstrate significant development are moved to Tier 3 intervention (Jenkins et al., 2013; Vaughn and Swanson, 2015).

Reading difficulties have serious implications on students’ academic success and motivation, and there has been a growing body of research that evidences the need to develop early interventions that are tailored to the needs of each student to reduce the likelihood of more severe disabilities (Arias-Gundín and Llamazares, 2021) and decrease the differences between students (Pfost et al., 2014). Several studies support RtI effectiveness in the intervention in reading disabilities (e.g., Suggate, 2010; van Norman et al., 2020). In this study, we will focus specifically on Tier 2 reading interventions. A recent meta-analysis (Gersten et al., 2020) focused on the effects of Tier 2 reading interventions on first to third graders considered at-risk for reading disabilities. Most of the 33 analyzed studies included instruction on phonological awareness, decoding, oral reading fluency and spelling, but intervention in vocabulary and comprehension was seldom described. The results showed larger effect sizes for word or pseudoword reading, compared to reading comprehension and fluency. The observation of larger effects in foundational skills, such as decoding or fluency, compared to comprehension has been consistently reported in other meta-analyses focused on the intervention in reading difficulties in the first years of schooling (e.g., Wanzek et al., 2016). The meta-analysis by Gersten et al. (2020) also showed that interventions including phonological awareness training had significantly smaller effects than interventions without it, suggesting that spending time explicitly teaching phonological awareness is counter-productive after children start to develop decoding skills. Another important result was that various intervention characteristics (e.g., type of interventionist, group size) had little moderating influence on the intervention effects. Previous meta-analyses of the effects of Tier 2 type reading interventions in primary school students had also suggested that the positive effects observed do not depend on the number of intervention hours (Wanzek et al., 2016, 2018). Although these meta-analyses provided important insights on Tier 2 interventions in the first years of reading acquisition, one of their limitations was that they did not analyze whether digital tools were used as part of the intervention, or whether they included complementary homework or some type of remote intervention.

Particularly in the last decade, there has been a significant increase in the use of technology for assessment and intervention with learning disabilities (Dean et al., 2021). Several systematic reviews have shown that a wide diversity of technology is used in this field, from text-to-speech tools to computer-based software designed to improve specific skills (Chai and Chen, 2017; Wood et al., 2018; Dogan and Delialioglu, 2020). One of the most widely used computer programs to promote basic reading skills is GraphoGame, which has been adapted for about two dozen of countries (Ojanen et al., 2015; Ahmed et al., 2020; Dean et al., 2021; Lyytinen et al., 2021). A recent systematic review by McTigue et al. (2020) focused on the effects of GraphoGame on word reading skills. This review concluded that the effects were mostly small, but a more detailed review of the moderators suggested sizeable effects when there was a high degree of adult participation during the intervention. Some research suggests that new technologies can be successfully integrated in interventions in reading disabilities based on the RtI framework. For example, Duijnen (2021) describes a synchronous online fluency intervention with three struggling readers in second and third grade, with similar reading performance. The students were involved in an 8-week small group intervention, totaling 15 sessions of 45 min each. The results indicated a noticeable increase in word reading accuracy, decoding skills, and reading comprehension after the intervention.

The results of a recent systematic review on technology-based interventions for children with reading difficulties, including 45 studies published between 2010 and 2020, indicated that most interventions were multi-component; that is, they addressed more than one reading component (Alqahtani, 2020). The same review also indicated that in 25 studies (55% of the total) the students worked alone with the intervention tool, whereas in the remaining studies an adult was involved. This proliferation of technology in the intervention in reading disabilities has been accompanied by a concern of how they should be employed to maximize the likelihood of positive outcomes. A recent Delphi study aiming to gather guidelines for good practices in the use of technologies for intervention in dyslexia (Lorusso et al., 2021) suggested an overall positive attitude toward the use of ICT-based interventions, with the flexibility, engagement, and cost-effectiveness being pointed out as some of the advantages of the format. Moreover, the 18 experts involved in this study provided some insights on best practices: (a) the intervention should be started before the third grade and should last up to 6 months; (b) the intervention should target phonological awareness, visual abilities, lexical skills, and grapheme-to-phoneme conversion; and (c) the families’ compliance and their ability to support the children and to mediate and supervise the completion of ICT-based activities should be considered (Lorusso et al., 2021).

Social validity refers to the perceptions of the participants in an intervention regarding its goals, procedures, and effects (Wolf, 1978; Foster and Mash, 1999). These perceptions are vital given that they may foster (or hinder) intervention sustainability: if participants and other actors involved, such as teachers and parents, perceive the intervention as important, worthwhile, easy to implement and enjoyable, they will be more likely to adhere to it and intervention will be more likely to go on (Kozleski et al., 2021). However, social validity in reading interventions has seldom been explored. A review by Lindo and Elleman (2010) focused on studies on reading interventions published between 2000 and 2006. They reviewed more than 600 studies and found that only 14 studies included data on social validity, and these were focused either on students’ (*n* = 4) or teachers’ (*n* = 10) perceptions. Parents’ perceptions have not been addressed, although they have been involved in some Tier 2 reading interventions, either as an active part in the intervention or in a less active role, as supporters in additional activities carried at home (Gerzel-Short, 2017; Grindle et al., 2019). Moreover, as suggested in the previously referred Delphi study (Lorusso et al., 2021), the families’ compliance is a crucial factor for the success of ICT-based reading interventions and, this depends to a large extent on how important, feasible and effective they think the intervention is.

### The Present Study

The outbreak of the COVID-19 pandemic hastened the need to integrate ICT in interventions for a wide range of disorders. Across the world, lockdowns, illness, quarantine, and prophylactic isolation have limited the access of children to education and intervention services. Consequently, there were some attempts to create interventions that could be delivered remotely to children with learning disabilities. However, most of these consisted of delivering traditional interventions using programs such as Zoom for synchronous remote communication (e.g., Alves and Romig, 2021; Beach et al., 2021; Cruz et al., 2021). In this study, the intervention started face-to-face in November of 2020 but was shifted to a remote modality between January and March of 2021 due to lockdown. We conducted a single-group study with pre-test and post-test and used an e-learning platform for interventions in reading disabilities called “I’m still learning” (Ribeiro et al., 2016). This tool includes tasks for the assessment and intervention in primary school children with, or at-risk for, reading disabilities, focusing specifically on phonological awareness, word reading, oral reading fluency and comprehension. Because this study was conducted with second-grade students, the phonological awareness intervention tasks were discarded. Some of the tasks can be performed independently by the students, but for others, the guidance of an adult is required (e.g., to transition between tasks and to provide more specific feedback). As indicated before, studies that address the social validity in reading interventions are scarce. Although, the intervention addressed in this study was delivered by professionals, parents had a supporting role in the part of the intervention that was conducted remotely. Therefore, besides directly assessing the effects of the intervention in students’ abilities, parents’ perceptions were also addressed to collect evidence of social validity for the remote intervention, supported by the digital tool, and conducted out of school ours. Therefore, the goals of this study were: (a) to explore the existence of gains in students’ word reading, oral reading fluency and listening comprehension at the end of a Tier 2 intervention performed using the referred digital tool; (b) to assess whether the gains depended on the students’ gender, the number of sessions attended and the interventionist; and (c) to investigate parents’ perceptions regarding the remote intervention with their children using the digital tool.

## Materials and Methods

### Participants

The sample comprised 81 second-graders who were flagged as being at-risk for reading disabilities in a universal screening (*N* = 528) conducted in a municipality from the North of Portugal. This universal screening was performed at the beginning of the school year in 27 public schools, comprising 29 classes, and included assessments of letter recognition, reading fluency, and listening comprehension (Santos et al., 2020b). All assessments were administered by trained teachers in a classroom setting. Students who scored below the cutoff scores in the universal screening (<3 points in oral reading fluency and/or ≤7 points in listening comprehension) were flagged as being at-risk. All the selected students had fluency deficits and 21 also had concurrent listening comprehension deficits. Only children who recognized letters and who were, at least already capable of identifying words composed of simple CV syllabic structures (consonant + vowel) where included in this sample. Children who did not demonstrate these skills were referred to (and later supported by) other school services. Regarding gender, 36 (44.4%) were boys and 45 (55.6%) were girls. Students were aged between 6 and 8 years old (*M* = 6.95; *SD* = 0.391) and were not engaged in any other intervention or additional support for learning in the school.

### Measures

#### Test of Word Reading (*TLP; Teste de Leitura de Palavras*)

The TLP (Chaves-Sousa et al., 2017a,b) is a standardized test comprising four vertically scaled test forms for students in grades one to four to evaluate word reading. The test forms TLP-1 and TLP-2 were used in this study. Each test version includes 30 single words that are displayed consecutively, in a randomized order, *via* a computer application. The test administration is untimed. During the test application, word reading accuracy (correct/incorrect) is recorded in the platform by the evaluator. The raw scores (total number of words read correctly) are then converted to a standardized (scaled) score. The standardized scores are in a scale with a mean of 100 and standard deviation of 10. The expected mean standardized score is 100 (*SD* = 10) at the end of the first grade and 109 (*SD* = 10) at the end of the second grade. The test has adequate indicators of reliability and validity (Chaves-Sousa et al., 2017a).

#### Test of Listening Comprehension of Narrative Texts (*TCTMO-n; Teste de Compreensão de Textos na Modalidade Oral-Narrativo*)

The TCTMO-n (Santos et al., 2015; Viana et al., 2015) is composed of four vertical scaled test forms to assess students’ listening comprehension from first to fourth grades. The test forms TCTMO-n-1 and TCTMO-n-2 were used in this study. Each test form includes four texts. Students heard the recorded narrative texts followed by 30 multiple-choice listening comprehension questions, presented in the same format. The questions had three alternatives (one correct). Questions assessed literal comprehension, inferential comprehension, reorganization, or critical comprehension (Català et al., 2001). The test administration is untimed, and the total number of correct answers is computed and converted to a standardized score. The standardized scores are in a scale with a mean of 100 and standard deviation of 10. The expected mean standardized score is 100 (*SD* = 10) at the end of the first grade and 106 (*SD* = 10) at the end of the second grade. High reliability and evidence of construct and criterion validity has been provided for the test (Santos et al., 2015; Viana et al., 2015).

#### Test of Reading Fluency (*TFL; Teste de Fluência de Leitura*)

The TFL (Ribeiro et al., 2014) assesses oral reading fluency and consists of an unpublished narrative text that students are required to read aloud. The test administration is individual and has a time limit of 3 min. The number of reading errors is registered, and the mean number of words read correctly per minute is calculated.

#### Parents’ Questionnaire

A questionnaire was designed to assess parents’ perceptions of the remote intervention. This self-report questionnaire included 17 items that were answered using a 5-point Likert scale from 1 (Totally disagree) to 5 (Totally agree). The items were developed following the social validity dimensions proposed by Wolf (1978): (a) significance of the goals; (b) appropriateness of the procedures; and (c) importance of the effects. Therefore, the items assessed not only the perceived effects of the intervention, but also the suitability of the intervention and materials, the appropriateness of the methodology, the suitability of the schedule and equipment and the interventionists’ performance. One open response question was also presented so that parents could provide additional comments. The questionnaire can be consulted in the Supplementary Material.

### Procedures

This study was approved by, and conducted according to, the ethical recommendations of the Ethics Committee of the University of Minho. Authorization from the municipality and the school boards was also obtained. Additionally, before participating in data collection and intervention delivery we obtained informed consent forms for each student, signed by their parents/tutors. After the universal screening and before the intervention, students who scored below the cutoff scores were administered standardized measures of word reading, oral reading fluency, and listening comprehension (October–November 2020). These measures were also administered after the intervention (May–June 2021) by the same researchers who delivered the intervention. The first two measures were administered individually. The test of listening comprehension was administered in small groups. At the end of the remote intervention, parents were asked to respond to a questionnaire about their perceptions regarding the intervention carried out during the lockdown. This questionnaire was presented using Google Forms and accessed *via* a link sent *via* email. Although the participation was anonymous and all parents were invited to participate, only 49 parents responded.

### Intervention

A Tier 2, small group intervention (3–5 students), was organized to promote fluency and listening comprehension. Groups were organized based on the COVID-19 pandemic sanitary rules at the time, that determined that students from different classes could not mix inside the schools. Therefore, students in each intervention group were from the same class. The intervention incorporated activities from the “I am still learning” online platform. Sessions, each lasting approximately 40 min, occurred twice a week. The face-to-face intervention started in November 2020 and occurred outside the classroom, in a schedule agreed by the elementary school teacher, during the school day. Remote intervention occurred between January 21st until mid-March of 2021. In these remote sessions, the major divergences from the original intervention were that interventionists delivered the program using Zoom and sessions occurred mostly after school hours in a schedule agreed by parents. A mean number of 26.85 sessions (*SD* = 6.62; Median = 29; Minimum = 8; Maximum = 37). The structure of the intervention was similar for all groups. Parents were present during the remote intervention to help children access the link to participate in the session, and to supervise and observe the children’s performance. The intervention was delivered by three of the study authors (henceforth referred to as the interventionists). These interventionists are qualified educational psychologists with experience delivering reading interventions. Each session included an introduction to a new text. Texts had a short length–50 to 200 words–and its content was related to the children’s experiences like daily routines, animals, and family. The sessions were structured as follows:

(1) Activation of previous knowledge

Using the text title, students were asked to discuss what they thought the story was about and any personal experiences related to the content of the title. If the students had questions about some words, the meaning was discussed.

(2) Word reading training

A selection of complex words in each text–whether due to their low frequency or due to a complex phonological structure–is available on the platform. Students were trained to read these words using the digital platform: each word appeared individually on the computer screen and students heard the reading of the word while performing silent reading. The platform allowed the students to hear the words as many times as they desired. In a final step, all the words trained were randomly presented on the screen in a list format and students were asked to read them aloud. If the student made an error on any of these tasks, the interventionist provided the correct reading and the meaning of the words.

(3) Oral reading fluency training

First, the digital platform provided a model reading of the full text. Next, each student practiced reading the text or an excerpt from the text. This was first done *via* assisted reading (i.e., a recording playing in the platform and students reading silently; Rasinski, 2003), and later *via* independent reading (i.e., each student read the text out loud and the interventionist assisted if necessary and provided comments on the reading). Students were invited to practice the reading at home and the following session began with a new reading of the same text. The interventionist then gave each student feedback about their speed, accuracy, and prosody.

(4) Listening comprehension training

Students heard the recorded text again followed by multiple-choice listening comprehension questions. The questions, presented orally by the platform, had three alternatives (one correct). Questions considered literal comprehension, inferential comprehension, reorganization, or critical comprehension (Català et al., 2001). Students discussed the alternative they considered correct and the reasons for that choice. This choice was then recorded on the platform by the interventionist. After the questions were answered, the platform gave feedback by showing the list of the chosen alternatives and an indication of whether the answer was correct. The interventionists then discussed the results with the students and provided correct alternatives when the incorrect answer was given.

### Data Analysis

Six students were excluded from the analyses because they did not complete at least one of the measures at pre-test or post-test. The values of skewness and kurtosis were analyzed as indicators of a normal distribution: values lower than | 3| for skewness and | 7| for kurtosis indicated no robust violations to the assumption of normality (Hair et al., 2009). Independent samples *t*-tests were performed to test the differences in the standardized measures administered at pre-test between boys and girls, and the differences among the groups assigned to each interventionist. When the assumption of homogeneity of variances was not met, the Welch correction was applied. Cohen’s *d* was used as a measure of effect size: 0.20 suggests a small, 0.50 a medium, and 0.80 a large effect (Cohen, 1988). Three paired-samples *t* tests were computed to assess change students’ skills from pre-test to post-test. A standardized gain score was computed using the following equation: (post-test scores–pre-test scores)/standard deviation at pre-test. A Pearson correlation coefficient was estimated to assess whether gains in oral reading fluency were associated with students’ initial levels of word reading. Next, three multiple linear regression models were computed to assess the effects of the students’ gender, number of intervention sessions attended and interventionist on the gains obtained by the students in the three measures. Multicollinearity, independence and normality of residuals and the presence of severe outliers were checked prior to the analysis. Parents’ perceptions of the intervention were analyzed by computing the frequencies of the scores for each item of the questionnaire. Statistical analyses were performed using IBM^®^ SPSS Statistics 28.

## Results

### Student Outcomes

Table 1 presents descriptive statistics and the outcomes of the paired-samples *t* tests. The results indicate a significant increase in all skills from pre-test to post-test. The effect was large in all three variables, but oral reading fluency and word reading had the largest effect sizes.

**TABLE 1:** Differences in listening comprehension, word reading, and oral reading fluency between pre-test and post-test.

	T1	T2			
Outcome variable	M (SD)	M (SD)	*t* (df)	*p*	*d*
Listening comprehension	95.24 (9.09)	102.31 (9.08)	−6.789(74)	< 0.001	0.778
Word reading	80.97 (27.32)	104.20 (8.17)	−7.913(74)	< 0.001	1.152
Oral reading fluency	9.05 (9.98)	37.02 (18.30)	−17.279(74)	< 0.001	1.898

*M, mean; SD, standard deviation; T1, pre-test; T2, post-test.*

Regarding gender differences in the scores obtained in the pre-test, no significant differences were found in word reading, *t*(66) = −1.908, *p* = 0.061, *d* = 0.416, oral reading fluency *t*(73) = −1.154, *p* = 0.252, *d* = 0.268, or listening comprehension, *t*(73) = 1.231, *p* = 0.222, *d* = 0.286. Additionally, no differences were found between the groups assigned to each interventionist in any of the three variables measured in the pre-test: word reading, *F*(2,72) = 1.176, *p* = 0.314, _p_η^2^ = 0.032; oral reading fluency, *F*(2,72) = 0.287, *p* = 0.751, _p_η^2^ = 0.008; listening comprehension, *F*(2,72) = 0.781, *p* = 0.462, _p_η^2^ = 0.021.

Table 2 shows the results of regression analysis to assess whether gains depended on the children’s gender, the interventionist and the number of intervention sessions attended. None of the variables was a significant predictor of the gains in word reading, oral reading fluency or reading comprehension. The association between word reading in pre-test and gains in oral reading fluency was not statistically significant (*r* = 0.216, *p* > 0.05), suggesting that the gain did not depend on the initial level of word reading.

**TABLE 2:** Results of the regression models to predict standardized gains in listening comprehension, word reading, and oral reading fluency.

Independent variables	Model 1: Listening comprehension				Model 2: Word reading				Model 3: Oral reading fluency			
	R (R^2^)	β	*t*	*p*	R (R^2^)	β	*t*	*p*	R (R^2^)	β	*t*	*p*
Gender	0.215 (0.046)	0.014	0.117	0.907	0.251 (0.063)	–0.227	–1.913	0.060	0.117 (0.014)	0.071	0.583	0.562
Number of sessions		–0.157	–1.241	0.219		–0.093	–0.740	0.462		0.062	0.485	0.629
Interventionist (2)		0.149	1.189	0.239		–0.130	–1.047	0.299		0.095	0.750	0.456
Interventionist (3)		0.001	0.007	0.994		–0.122	–0.928	0.356		0.041	0.303	0.763

*To test for the effect of the interventionist, students assigned to interventionist 1 were the reference group.*

### Parents’ Perceptions

Table 3 presents the descriptive statistics of the responses to the parents’ questionnaire. Parents’ perceptions were particularly positive in the items related to the interventionist’s performance (answers ranging from 91.8 to 98% of responses agree or totally agree) and in the items related to the suitability of the intervention content, materials, and structure of the sessions (answers ranging from 81.6 to 93.9% of responses agree or totally agree). Nine parents did not consider that the duration of the intervention was adequate. Three of these indicated that the intervention should be longer, but the remaining six did not provide additional comments. Regarding the perceived effects of the intervention, most parents agreed that the intervention improved their children reading skills (83.7%) and learning (87.8%). Additionally, most parents (85.7%) did not find the remote intervention disruptive for the household activities and reported adequate access to the internet and computer availability for the student to perform the intervention tasks (89.8%).

**TABLE 3:** Frequencies of responses in each item of the parents’ questionnaire.

Items	Totally disagree	Disagree	Do not agree nor disagree	Agree	Totally agree
	
	*N* (%)	*N* (%)	*N* (%)	*N* (%)	*N* (%)
**Suitability of the intervention and materials**					
1. The intervention content was adequate	0 (0%)	1 (2.0%)	2 (4.1%)	21 (42.9%)	25 (51.0%)
2. The materials were adequate	0 (0%)	1 (2.0%)	2 (4.1%)	19 (38.8%)	27 (55.1%)
3. The duration of the intervention was adequate	0 (0%)	2 (4.1%)	7 (14.3%)	21 (42.9%)	19 (38.8%)
**Appropriateness of the methodology**					
4. The structure of the sessions was adequate	0 (0%)	0 (0%)	3 (6.1%)	20 (40.8%)	26 (53.1%)
5. My son/daughter was cheerful during the intervention sessions	1 (2.0%)	0 (0%)	1 (2.0%)	18 (36.7%)	29 (59.2%)
6. My son/daughter talked about the intervention with me and with his/her colleagues	2 (4.1%)	3 (6.1%)	8 (16.3%)	16 (32.7%)	20 (40.8%)
**Suitability of the schedule and equipment**					
7. The schedule of the intervention was not disruptive for family life	1 (2.0%)	1 (2.0%)	5 (10.2%)	16 (32.7%)	26 (53.1%)
8. It was possible to use adequately a computer and access internet	0 (0%)	2 (4.1%)	3 (6.1%)	18 (36.7%)	26 (53.1%)
**Perceived effects of the intervention**					
9. The intervention allowed my son/daughter to remind previous knowledge	0 (0%)	1 (2.0%)	9 (18.4%)	16 (32.7%)	23 (46.9%)
10. The intervention allowed my son/daughter to learn more	0 (0%)	1 (2.0%)	5 (10.2%)	21 (42.9%)	22 (44.9%)
11. The intervention allowed my son/daughter to improve his/her reading skills	0 (0%)	3 (6.1%)	5 (10.2%)	22 (44.9%)	19 (38.8%)
12. The intervention made my son gain the habit of studying at home	0 (0%)	3 (6.1%)	13 (26.5%)	17 (34.7%)	16 (32.7%)
**Interventionists’ performance**					
13. The interventionist organized the intervention adequately	0 (0%)	0 (0%)	1 (2.0%)	14 (28.6%)	34 (69.4%)
14. The interventionist was clear about the content to be learned in each session	0 (0%)	0 (0%)	2 (4.1%)	14 (28.6%)	33 (67.3%)
15. The interventionist clarified any doubts	0 (0%)	0 (0%)	1 (2.0%)	15 (30.6%)	33 (67.3%)
16. The interventionist’s work raised the interest of the students in the task	0 (0%)	0 (0%)	3 (6.1%)	15 (30.6%)	31 (63.3%)
17. The interventionist’s work contributed to the improvement of my son/daughter’s reading skills	0 (0%)	0 (0%)	4 (8.2%)	16 (32.7%)	29 (59.2%)

## Discussion

The first two goals of this study were to assess the gains in word reading, oral reading fluency, and listening comprehension at the end of a Tier 2 intervention performed using a digital tool, and to investigate whether the gains depended on the students’ gender, the number of sessions attended and the interventionist. The intervention included strategies to promote word reading skills and oral reading fluency, as well as vocabulary and listening comprehension. While most Tier 2 interventions in the early years of schooling have focused on the promotion of basic reading skills (Gersten et al., 2020), the inclusion of activities to promote linguistic skills is also vital given that these contribute directly to decoding and to reading comprehension (Cadime et al., 2017; Santos et al., 2020a).

Regarding the first goal, we found significant improvements for all three outcome variables after the intervention. The largest effect size was observed for fluency, followed by word reading. This finding aligns with previous literature that suggests higher gains in basic reading skills compared to comprehension in Tier 2 interventions in the first years of schooling (Wanzek et al., 2016; Gersten et al., 2020). This finding may reflect the higher focus of the interventions on these skills compared to the shorter time devoted to fostering vocabulary or listening comprehension (Gersten et al., 2020). Moreover, in our study, the intervention to foster listening comprehension encompassed strategies such as activation of previous knowledge, clarification of difficult words and response to questions and feedback, but did not include other strategies that research has shown to be effective, such as the training of cognitive and metacognitive strategies (Goh and Taib, 2006; Baker et al., 2020). Additionally, it is worth noting that the students participating in this study were in an early stage of reading acquisition, and all of them were experiencing difficulties in the automatization of reading. In the first years of schooling, phonics and fluency instruction are key components in literacy instruction in the classroom (Spear-Swerling et al., 2016) and research has suggested that struggling readers obtain the largest gains with systematic reading interventions (Suggate, 2010; Cruz et al., 2021). Therefore, the characteristics of our sample might have played a role in the large gains observed in word reading and oral reading fluency. Our results also shown that the gains observed in fluency did not appear to depend on initial levels of word reading. Although the absence of a control group prevents us from attributing the observed gains in reading directly to the intervention program, the results of this study are quite encouraging regarding the use of a digital tool to promote reading and linguistic skills in students at-risk for reading disabilities. It is noteworthy that the use of the tool did not totally replace the necessity for an interventionist. Although some of the tasks could be completed independently by the students, our option was to use the tool as part of a more structured intervention supervised and guided by the interventionists. Although there is a large variation in the degree of independency provided by the ICT-based interventions (Alqahtani, 2020), research is suggestive of higher gains when there is some amount of involvement of adults when using digital tools to foster children’s reading skills (McTigue et al., 2020). Regarding the second goal, the results of our study showed that the gains did not depend on the children’s gender, the number of sessions attended and the interventionist. This finding is similar to the ones reported in meta-analyses that analyzed the effects of interventions with children with reading disabilities (Wanzek et al., 2016, 2018; Gersten et al., 2020).

The third goal of this study was to investigate parents’ perceptions regarding the remote intervention with their children. Parents’ perceptions were quite positive regarding its content, structure, and materials used, including the digital tool. However, some parents indicated that the duration of the intervention was not sufficient. Parents’ perception of the necessity of more hours of intervention may be influenced by the acknowledgment that, although their children experienced significant intraindividual gains, they were still performing below grade level in reading. As an example, in oral reading fluency, the mean number of words read correctly per minute by the students at the end of the intervention was 37.02 (*SD* = 18.30), a number well below the 90 words indicated as a reference in the curricular benchmarks for Portuguese language in the second grade (Buesco et al., 2015). The finding that students with, or at-risk of, learning disabilities can make large gains when they are provided intensive and systematic intervention, yet still lag behind their peers, has been widely reported in the literature (Gilmour et al., 2019), but research has also suggested that early interventions can contribute to reduce this gap (Wanzek et al., 2018). Overall, the findings of our study provide support for the social validity of the intervention. Although evidence of social validity is not in itself evidence of efficacy of an intervention, it involves dimensions that can contribute to efficacy, such as acceptability and viability (Foster and Mash, 1999). In this case, the data collected from parents suggest that the intervention is viable and will be accepted in a particular setting, namely, in a remote modality where students receive the intervention when at home. The effects of remote interventions with children have been a recent concern in research, mainly due to the disruption induced by the COVID-19 pandemic. Overall, the research results suggest not only positive effects in the targeted skills (e.g., Duijnen, 2021), but also high acceptability and positive perceptions of feasibility and effectiveness of remote interventions with children with reading disabilities or other neurodevelopmental disorders (Beach et al., 2021; Su et al., 2021). Our study adds to this body of research showing also positive perceptions of a remote intervention supported by a digital tool to promote reading skills in students facing reading difficulties.

The main limitation of this study was the absence of a control group, which precludes us from concluding that the observed gains are directly attributable to the intervention. Also, the intervention groups were not randomly assembled. Therefore, the generalization of results should be made with caution. Another limitation is related to the procedures used to assess the intervention fidelity. Although the use of a standardized online platform potentiates the likelihood that the intervention was administered as intended, other procedures to assure intervention fidelity, such as observations and ratings (King-Sears et al., 2018), were not used. Future studies should consider the implementation of these techniques to assess fidelity. A fourth limitation is related to the limited information gathered on the social validity of the intervention. Only parents’ perceptions were collected, using a questionnaire. Future studies should also address students’ and teachers’ perceptions and use complementary methods such as interviews or focus groups, that allow a more in-depth exploration of how these groups perceive the relevance of the goals, the feasibility of the procedures and the effects of the intervention. A final limitation was that no information regarding families, such as socioeconomic status, was collected. Future studies should address whether the intervention effects or the parents’ perceptions vary as a function of the families’ characteristics.

Nonetheless, the results provide some important insights on the use of ICT and digital tools in reading interventions. Firstly, our tool was easily integrated both in the face-to-face and in the remote intervention phases, following the guidelines for best practices in the use of ICT in intervention in reading disabilities (Lorusso et al., 2021). Specifically, the parents were involved in the intervention sessions and granted access to the digital tool. Only a few parents reported that the intervention was disruptive for the household. Therefore, our results suggest that the use of digital tools in a remote intervention modality can be a feasible alternative to address the needs of students with reading disabilities during a lockdown or prophylactic isolation in a context of a pandemic, if granted access to a computer and an internet connection. Finally, this study suggests that the intervention presented was effective with struggling readers, combining a RTI framework and the use of digital technologies.

## Data Availability Statement

The raw data supporting the conclusions of this article will be made available by the authors, without undue reservation.

## Ethics Statement

The studies involving human participants were reviewed and approved by Ethics Committee of the University of Minho. Written informed consent to participate in this study was provided by the participants’ legal guardian/next of kin.

## Author Contributions

IC, IR, FV, and SS: conception and design of the study. JC, MC, and DM: data collection and intervention. IC and SS: statistical data analyses. All authors were involved in interpreting and discussing the results and in drafting the manuscript and revising it critically for important intellectual content.

## Conflict of Interest

The authors declare that the research was conducted in the absence of any commercial or financial relationships that could be construed as a potential conflict of interest.

## Publisher’s Note

All claims expressed in this article are solely those of the authors and do not necessarily represent those of their affiliated organizations, or those of the publisher, the editors and the reviewers. Any product that may be evaluated in this article, or claim that may be made by its manufacturer, is not guaranteed or endorsed by the publisher.
