# Supplementary material for: An Intervention in Reading Disabilities Using a Digital Tool During the COVID-19 Pandemic
Source: Front Psychol. 2022 May 6;13:862383. doi: 10.3389/fpsyg.2022.862383 (PMC9120957; doi:10.3389/fpsyg.2022.862383)
Supplement: Supplementary file 1 [file Table_1.DOCX]

**QUESTIONÁRIO**

Este questionário tem como objetivo avaliar a intervenção realizada a distância. As respostas a este questionário são, por isso, muito importantes para a melhoria da intervenção em situações similares.

1. O que foi trabalhado era adequado às caraterísticas do/a meu/minha educando/a.
2. Os materiais utilizados foram adequados e do agrado do/a meu/minha educando/a.
3. O tempo que durava a intervenção foi adequado.
4. A maneira como as sessões foram organizadas foi adequada.
5. O/A meu/minha educando/a mostrava-se satisfeito/a nas sessões de intervenção.
6. O/a meu/minha educando/a falava com colegas e connosco sobre o trabalho que realizava.
7. O horário foi adequado à organização da vida familiar.
8. A utilização do computador e da internet foi possível e adequada.
9. A intervenção permitiu ao/à meu/minha educando/a relembrar conhecimentos anteriores.
10. A intervenção permitiu ao/à meu/minha educando/a aprender melhor o que foi ensinado.
11. A intervenção permitiu que o/a meu/minha educando/a melhorasse ao nível da leitura.
12. A intervenção permitiu que o/a meu/minha educando/a criasse o hábito de estudar em casa.
13. A mediadora organizou bem a intervenção.
14. A mediadora era clara relativamente ao que ia ser trabalhado.
15. A mediadora esclarecia as dúvidas que eram colocadas.
16. A intervenção da mediadora contribuía para o interesse dos alunos sobre o que estavam a aprender.
17. O trabalho da mediadora contribui para que o/a meu/minha educando/a melhorasse ao nível da leitura.
